# Contextualizing Caregiver Burden in Mild Cognitive Impairment: A Dyadic Perspective

**DOI:** 10.3390/ijerph22111656

**Published:** 2025-10-31

**Authors:** Emily L. Giannotto, Christopher Hertzog, Amy D. Rodriguez

**Affiliations:** 1 Department of Neurology, School of Medicine, Emory University, Atlanta, GA 30322, USA; 2 School of Psychology, College of Sciences, Georgia Institute of Technology, Atlanta, GA 30332, USA; christopher.hertzog@psych.gatech.edu; 3 Atlanta VA Center for Visual and Neurocognitive Rehabilitation, Decatur, GA 30033, USA

**Keywords:** mild cognitive impairment, caregiving, caregiver burden, depressive affect, stress, mutuality, cognition, sleep quality, daily diary, dyadic analysis

## Abstract

Multidimensional approaches to understanding the daily lived experiences and well-being among spousal dyads, where one partner has diagnosed mild cognitive impairment (MCI) and the other serves as an informal caregiver, is a relatively unexplored area of research. This study examined contextual day-to-day patterns of spousal dyads’ caregiver burden, depressive affect, stress, relationship mutuality, sleep, and cognition from the perspective of both dyad members. For 14 consecutive nights, 27 dyads (*n* = 54 individuals) completed online daily diary forms. The forms included self and informant reports about daily caregiver burden, depressive affect, stress, dyadic interactions, memory, and sleep quality. Exploratory multilevel modeling was performed to understand how daily fluctuations among these aspects of everyday living for both dyad members were associated. Mutuality emerged as an important moderator for caregiver burden and depressive affect outcomes, underscoring the significance of the relationship between care recipients with MCI and their caregivers. Sleep debt was also associated with contagion effects among partners’ depressive affect, stress, mutuality, and cognition. The present study demonstrates the value of multifaceted investigations that account for contextually relevant factors using daily repeated measures with both dyad members to better understand the MCI caregiver experience. Larger, more diverse samples are needed for generalizability of findings.

## 1. Introduction

Caregiving is an intrinsic aspect of relationships that involves caring for the affective, behavioral, and practical well-being of another [1]. Transitioning into a caregiving role involves changes to the nature of the relationship as well as increased care responsibilities [1,2,3]. We focus on the dynamic of caring between older couples where one member of the dyad has been diagnosed with mild cognitive impairment (MCI) and the other serves as an informal caregiver. Given that spouses’ lives are inextricably linked, changes in daily living as a consequence of cognitive function within one partner impacts the lived experience of the other [4]. In the present work, we account for daily functioning and contextual factors involved in everyday life for people with diagnosed MCI and their spousal caregivers.

Caregiving in MCI is unique in that care recipients experience impaired cognition in one or more domains (e.g., memory, language) but do not have dementia and largely maintain their ability to complete the instrumental activities of daily living (IADLs) [5,6]. Essentially, individuals with MCI can navigate most aspects of daily life, but they may need assistance with more complex tasks. Currently, an estimated 6% of the US population between 70 and 89 are diagnosed with MCI each year [7,8]. In addition, the rate of progression from MCI to dementia ranges between 5 and 17% and is higher for those who will go on to develop Alzheimer’s Disease (AD) [5,9,10,11,12,13,14]. Importantly, the prevalence of diagnosed individuals also represents the emergence of informal caregiving (i.e., unpaid spouses, family members) which is accompanied by a unique set of circumstances and changes that not only represents their care recipient’s cognitive decline but also their own lives as caregivers through MCI and potentially into dementia [15]. Importantly, caregiving in AD requires a higher level of care compared to MCI, whether formal or informal. Understanding MCI, caregiving, and the nature of spousal dynamics are integral aspects of properly contextualizing everyday outcomes for MCI caregivers.

### 1.1. Conceptualizing Burden in MCI Caregiving

Prior research indicates that caregiver burden can be significant for spousal MCI caregivers [16,17,18,19]. The burden and impact of supporting persons with MCI is potentially important but at present is under-researched [20,21,22,23]. Supporting a loved one with MCI implies transitioning into a caregiving role and involves increased responsibilities and emotional components that can contribute to caregiver burden [2,16,21,22,24,25]. Caregiver burden encompasses affective, psychological, social, financial, and physical components that may be associated with caregiving [26,27,28,29] and are often categorized as care recipient characteristics, caregiver characteristics, and the caregiving context [30,31]. However, caregiver burden is often conceptualized differently across studies [32] as subjective stress, emotional and physical strain, subjective burden, or objective burden [22]. The variety of ways that caregiver burden in MCI is conceptualized speaks to the complex nature of the stresses involved with MCI caregiving, such as physical health problems, perceived task or emotional load increase, feelings of partner loss or loneliness, and dealing with subjective stress. Additionally, spouses may attempt to increase collaboration in everyday life (i.e., goals and tasks) as a way to compensate for their partners’ impairments which may serve as another source of distress [4,33]. One study found that more than 35% of MCI caregivers reported clinically significant burden and that care recipients’ behavioral problems—not depression or cognition—were most strongly associated with this burden [28].

Higher caregiver burden has been associated with cognitive and emotional symptoms reported by both individuals with MCI and their informants [34]; MCI caregiver burden and depression has been associated with increased ADL impairment and problematic behaviors in the care recipient [35]. Other factors related to increased MCI caregiver burden include higher care recipient dependence on caregivers, e.g., [36], and anosognosia—the lack of awareness of one’s cognitive impairment [37]. Finally, another study found that depressive affect among caregivers was worse when they reported higher perceived burden, were more bothered by MCI, had poorer personal health, less social support, used more coping strategies, and had less knowledge about dementia [24]. Taken together, these findings support the importance of considering the care recipient’s cognition, affect, and behaviors when assessing the caregiver’s experiences. The current study was specifically designed to evaluate the multifaceted affective, behavioral, relational, and psychological experiences of both MCI caregivers and their care recipients within the context of everyday life.

Affection, responsibility, and care dynamics often shift as the person with MCI becomes more impaired, leading to changes in the relationship as the caregiver assumes more primary responsibilities [1,38]. The caregiving stress process model [1] has been referenced throughout the literature as a basis for understanding the complex nature of caregivers’ stress load as a major contributing component of caregiver burden. Stressors are defined as conditions, experiences, and activities that are problematic for caregivers and that fatigue, defeat, dampen their efforts, and threaten them in some way. The stress process model also accounts for caregiver demographics, characteristics, caregiving history, and access to resources through primary and secondary stressors. Primary stressors include the demands and magnitude of care that encompasses the needs and limitations of the care recipient, whereas secondary stressors include the role and psychological strains. Coping serves as a mediator and includes situational management, finding meaning to reduce threat, and stress management. Social support is another mediator, acting as a buffer against secondary stressors. Like Savla and colleagues [39], we used the caregiving stress process model framework to serve as a basis for conceptualizing complex relationships among daily stressors and mediating factors (e.g., coping and social support) involved in MCI spousal dyadic caregiving. However, the present work expands on Savla and colleagues’ study in three important ways. First, we added new methodology by including data from both dyad members. Second, we explored new analyses investigating caregiver outcomes through concurrent daily associations and those that occur from one day to the next (i.e., lagged associations). Third, we extended the data collection period to be longer. This novel approach allowed us to investigate contagion effects in more depth. Additionally, we considered different factors that influence caregiving, including behavioral problems associated with cognitive impairment and internal states associated with difficulty of daily caregiving. Care recipient behaviors represent indirect routes through which caregiver burden may or may not be experienced (e.g., caregivers may not be able to change these behaviors or may not observe them in the care recipient). Measuring behavioral problems does not provide information about the caregiver’s direct experience. Measuring subjective, internal states is needed to understand the caregiver’s affective experience. A novel aspect of this investigation is considering caregiving through the lens of internal states.

### 1.2. Spousal Dyads

The nature of spousal couples’ relationship dynamics is made even more complex during the transition to cognitive impairment and the experiences that take place thereafter. Drawing on socioemotional selectivity theory, spousal affect becomes interconnected over time through shared experiences and resultant meaning from the relationship itself [40]. Compared to other caregiving relationships, spousal dyads are often very different because of the underlying romantic relationship that is often in place long before the presence of cognitive impairment. Spouses also effect one another’s affect, mental health, and well-being [4,39,41,42,43,44,45,46,47]. Spouses dealing with MCI often have significant histories and highly interconnected experiences into older adulthood through shared environments, daily living, and collaborative problem solving.

These relationship dynamics and experiences among partners can be understood through mutuality—encompassing factors such as love, reciprocity, shared experiences, and values [48]. In the context of everyday living, spousal dyads may experience mutuality in the form of recalling shared memories, performing tasks, or expressing sentiments together [49]. Interestingly, older adult spouses have shown better collaborative recall performance when communication was positive or encouraged recall persistence. When communication was negative during the recall process, remembering was less collaborative but recall performance was not impacted [50]. Moreover, a caregiving spouse of someone with MCI may also be responsible for initiating or executing cognitively demanding tasks, intentions, or actions that at one-point were the sole responsibility of the care recipient, potentially impacting mutuality. The requirements and contexts vary but daily goal and task accomplishment often necessitates remembering that is at least in part shared among spouses. Importantly, everyday life circumstances may result in increased daily responsibilities for caregiving spouses and could be a contributing factor to daily caregiver burden with potential impacts on mutuality [51]. The present study specifically sought to understand mutuality among other factors such as daily caregiver burden, depressive affect, stress, and cognition among spousal dyads impacted by MCI.

#### 1.2.1. Well-Being, Affect, and Stress in Spousal Dyads

Contagion effects can also occur between spouses wherein they experience synchrony on a variety of indicators of well-being including depressive affect, perceived health, and meaning and purpose in life trajectory ratings [42]. These effects can also occur when negative affect and daily stress experienced by one partner negatively induces stress in the other [47,52,53]. For example, a study of older adult couples reported that a partner was more likely to endorse depressive symptomology if their spouse did as well [54]. In contrast, reduced negative affect in both partners has been associated with collaborative problem solving among couples on shared goals [55]. Finally, spouses who reported high marital satisfaction showed daily coupling of stress reactivity biomarkers, with a stress-buffering effect among couples who reported higher spousal support [56].

Moreover, stressors and stress reactivity may be shared among spouses. For example, collaborative coping involving communicative problem solving on shared stressors has been associated with higher negative affect among both spouses who were dealing with an ongoing health condition in one of the partners [57]. Additionally, higher caregiver burden and family conflict ratings have been associated with higher depressive affect among spouses, e.g., [17,31,58]. Savla et al. [39] conducted a weeklong diary study with spousal MCI caregivers to investigate daily stress, strain, spousal interactions, daily activities, well-being, sleep, and associated behavioral issues. They found that positive daily spousal interactions were associated with higher daily positive affect within-persons. On days when negative spousal interactions occurred, caregivers reported more negative affect. Further, care recipient behavioral problems were associated with same-day negative affect and salivary cortisol indicators of increased stress in the caregiver. These findings suggest that a broad range of factors can influence caregivers’ experienced stress–ranging from daily activities and spousal interactions to behavioral issues related to MCI both between and within individuals in spousal dyads.

Other daily diary studies have found that on days when healthy older adults reported interpersonal stressors, they also reported more memory failures that same day and the next day [59,60]. Spousal dementia caregivers who reported lower marital satisfaction simultaneously reported higher caregiver burden [61]. Aspects of well-being, depressive affect, stress, relationship dynamics, and caregiver burden significantly impact individuals’ and spousal dyads’ daily lived experiences. The current study sought to extend these findings by including daily measures of caregiver burden, depressive affect, stress, dyadic interactions, cognition, and sleep for both members of spousal dyads over a period of two weeks.

#### 1.2.2. Sleep

Sleep quality also impacts daily functioning including aspects of cognition and well-being [62,63,64,65]. For example, sleep disturbances can be exacerbated by mental health problems that may in turn impair circadian rhythms and emotional regulation [66]. Decreased sleep duration and sleep quality show increases with age and impact sleep fragmentation (the number of times adults wake up during the night), sleep disturbances, and the number and quality of deep restorative slow-wave sleep (SWS) stages [64,67,68,69,70,71]. Similarly, self-reported night-to-night sleep quality and average within-person sleep duration deviations have been associated with day-to-day cognitive performance variations as well as depressive affect across a range of tasks in middle aged and older adults [72,73,74,75,76]. However, middle aged and older adults appear to be less impacted by a poor night of sleep on next-day cognitive performance compared to younger adults [77,78,79]. The present study sought to address a critical gap in understanding how spouses’ sleep patterns may influence one another’s sleep quality, depressive affect, stress, functioning, and cognition in daily life.

A systematic review [80] found that sleep disturbance prevalence rates, which included sleep disorders, ranged from 7.9 to 49% for persons with MCI. Among persons with MCI, sleep disturbance has been associated with depressive affect, decreased cognitive performance, and worse physical health [81]. Moreover, persons with MCI show decreased SWS and greater sleep disturbance compared to cognitively healthy older adults which results in decreases in daytime alertness [81,82,83,84]. Given that many spouses share a bed, it is likely that greater sleep disturbance among persons with MCI directly impacts the sleep and/or functioning of the caregiver with whom they share a bedroom. Additionally, a two-week diary and sleep actigraphy study of persons with MCI found that sleep fragmentation, based on the amount time spent awake after falling asleep and the number of wakeups, was associated with decreased attention and executive functioning [85].

Sleep disturbance is also highly prevalent among caregivers and is often accompanied by caregiver burden, depressive affect, and worse physical health [86,87,88,89]. Caregivers not only report sleep disturbances and trouble falling asleep, which can be associated with their care recipient, but they also report struggling with energy and feeling tired throughout the day [90]. For example, compared to non-caregivers, informal dementia caregivers reported worse sleep quality ratings, next-day fatigue, and more variability in night-to-night sleep patterns during a weeklong diary study with accompanying concurrent objective sleep measurement [91]. Moreover, in an actigraphy-based sleep study with dementia caregivers, depressive affect was associated with lower sleep quality for older caregivers with lower self-rated health, they reported spending more time in bed but not more time asleep [92]. Finally, more hours per week spent caregiving among informal caregivers was associated with more self-reported sleep disturbance and lower quality of life ratings [93]. Sleep disturbance among memory impaired persons and their caregivers impacts individuals’ lives in meaningful ways. The interactive effect of sleep on daily cognition, affect, stress, and caregiver burden between spousal partners impacted by MCI has yet to be examined by accounting for both partners’ daily experiences and dyadic interactions. The present study addresses this gap.

### 1.3. Study Overview

Spousal MCI caregivers are impacted by everyday life occurrences that happen within the individual and the dyadic context such as sleep, stress, depressive affect, dyadic interactions, and spousal contagion. There has been little comprehensive work on examining multiple concurrent aspects of spousal daily living and well-being within the context of MCI caregiving dyads based on the caregiving stress process model. The present study used a two-week daily diary form to collect self- and informant reports on a wide range of personal and contextual variables that are known to impact dyadic daily lived experiences in one or both members of spousal dyads composed of one care recipient with diagnosed MCI and one identified caregiver.

The present study is novel in several ways that are important for advancing MCI caregiving research. First, the method of data collection is unique. Data were captured over 14 consecutive days on both members of the dyads to account for within-person and within-dyad level variation. The use of repeated measures within-persons and within-dyads over two weeks provides insight into variability in caregiver burden, depressive affect, stress, spousal interactions, cognition, and sleep within-persons and within-dyads across days (concurrent associations) and from one day to the next (lagged associations). Second, similar studies have typically only collected data from either persons with MCI or MCI caregivers, not both dyad members. Additionally, data from the course of each day from one dyad member has not typically been included in outcome analyses for the other partner. The current study does both. Third, to our knowledge, no other studies have collected such extensive repeated measures variables concurrently from both dyad members. Finally, the present study includes a unique range of measures that capture contextual holistic insights about potential daily influences on MCI caregiver burden sleep, stress, depressive affect, cognition, and spousal interactions.

#### Research Aims and Hypotheses

We were specifically interested in the patterns of association among aspects of daily cognition, well-being, and relationship dynamics through concurrent and lagged analyses. Our aims are framed to better understand spousal MCI caregivers’ (1) caregiver burden as a consequence of the daily MCI caregiving role via problematic behaviors exhibited by the care recipient and subjective ratings of the difficulty of caregiving as well as (2) affective well-being via depressive affect.

Study Aim 1: Concurrent Associations

We sought to explore daily caregiver burden, depressive affect, stress, spousal interactions, cognition, and sleep in MCI care recipient–caregiver dyads within days. We investigated how fluctuations among these variables influenced caregiver burden and depressive affect outcomes among caregivers across days.

Aim 1A: We examined caregiver burden difficulty as the outcome using concurrent analyses.

**Hypothesis** **1A.** *We hypothesized that higher daily depressive affect and stress in the caregiver, lower dyadic interaction ratings, lower care recipient memory ratings, and poorer caregiver sleep quality would be associated with higher caregiver burden difficulty within days.*

Aim 1B: We examined affective well-being with daily depressive affect in the caregiver as the outcome.

**Hypothesis** **1B.** *We hypothesized that the presence of problematic behaviors in the care recipient, higher care recipient depressive affect and stress, negative dyadic interaction ratings, and lower caregiver sleep quality would be associated with higher depressive affect in the caregiver within days.*

Study Aim 2: Lagged Associations

We sought to investigate lagged effects, specifically day-to-next-day effects of caregiver and care recipient sleep quality, and caregiver stress on caregiver burden and depression outcomes.

Aim 2A: We examined caregiver burden difficulty as the outcome using lagged analyses.

**Hypothesis** **2A.** *We hypothesized that higher daily depressive affect and stress in the caregiver, lower dyadic interaction ratings, lower care recipient memory ratings, and poorer caregiver sleep quality would be associated with higher caregiver burden difficulty from one day to the next.*

Aim 2B: We examined affective well-being with daily depressive affect in the caregiver as the outcome using lagged analyses.

**Hypothesis** **2B.** *We hypothesized that the presence of problematic behaviors in the care recipient, higher care recipient depressive affect and stress, negative dyadic interaction ratings, and lower caregiver sleep quality would be associated with higher depressive affect in the caregiver from one day to the next.*

## 2. Materials and Methods

### 2.1. Participants

Participants were recruited from the Charlie and Harriet Shaffer Cognitive Empowerment Program (CEP) at Emory University. The CEP is a research program that includes therapeutic activities for persons with diagnosed MCI and their caregivers. Twenty-seven dyads (*n* = 54 persons) were enrolled in this study. Dyads consisted of one care recipient with clinically diagnosed MCI due to Alzheimer’s Disease or a related disorder from the Emory Cognitive Neurology Clinic or the Emory Goizueta Alzheimer’s Disease Research Center (*n* = 27) and one (spousal or romantic) caregiver (*n* = 27). CEP participants must also be able to speak English, participate in group programming without severe behavioral disturbance, and ambulate and toilet independently. Both dyad members were required to share a residence, and consent and participate together in the present study. Prospective participants were recruited in-person at the CEP, via email or phone call and provided verbal consent prior to participation in study procedures. We attempted to contact 67 dyads for study recruitment. Of the 56 dyads we reached, 21 declined participation, 6 were excluded due to one dyad member declining participation, and three withdrew before scheduling began, resulting in a final sample size of 27 dyads. Study participation was voluntary, and participants did not receive compensation. The study protocol was approved by the Georgia Institute of Technology Institutional Review Board.

The average relationship duration for dyads was 41 years (SD = 16.32). Twenty-five dyads were married couples, two dyads were cohabitating long-term romantic partners including one same-sex couple. Care recipients were between 51 and 90 years old (M = 74.6, SD = 8.26, 10 females). The average care recipient TICS score was 29.81 out of 41. Caregivers were also between 51 and 90 years old (M = 71.4, SD = 8.35, 18 females). The average caregiver TICS score was 36.19 (SD = 2.96). Baseline demographic information for care recipients and caregivers is provided in Table 1 below.

### 2.2. Measures

#### 2.2.1. Nightly Diary Forms

Nightly self-report “diary” forms were completed by participants for 14 consecutive days. The nightly forms were adapted based on earlier work [94,95,96] that used similar diary-style self-reports. Care recipients and caregivers each completed their own form daily throughout the study period. The diary form included ratings of the following constructs: caregiver burden, depressive affect, stress, dyadic interactions, global memory, and sleep quality. Diary form questions were the same for care recipients and caregivers with the exception of informant reports of caregiver burden, which were only included in the caregiver version of the form. The diary was estimated to take between 10 and 15 min to complete and is described below.

##### Caregiver Burden

Caregivers were asked about caregiver burden in two ways. First, they completed seven yes or no questions from the Revised Memory and Behavior Problems Checklist (RMBPC) [97]. The RMBPC is an informant report of potential problematic behaviors (e.g., asking the same question over and over again) exhibited by persons with memory impairment. The RMBPC was scored out of seven possible points with higher scores indicating more problematic behaviors related to memory impairment that day. They were also asked about the difficulty of caring for their partner with MCI that day (e.g., Helping to care for my partner with Mild Cognitive Impairment was difficult today) on a sliding scale from 0 (“Not at all”) to 100 (“Very Difficult”). Higher scores indicated more difficulty associated with caregiving that day.

##### Depressive Affect

Participants completed the four-item version of the Geriatric Depression Scale (GDS-4) [98] each day with directions to answer the questions based on how they felt that day. The GDS-4 comprises yes or no questions (e.g., Are you basically satisfied with your life?) scored out of 4 possible points with higher scores reflecting a higher likelihood of depressive affect.

##### Stress

Participants indicated the amount of stress they experienced that day (e.g., How much stress did you experience today?) using a sliding scale from 0 (“None”) to 100 (“Extremely high”).

##### Dyadic Interactions

Participants completed six questions from the Mutuality Scale [48] about their relationship with their partner that day (e.g., How often do the two of you laugh together?) Questions were rated from 0 (“Not at all”) to 4 (“A great deal”). Responses were averaged across the questions to create a single daily mutuality score between 0 and 4. Higher scores indicated increased mutuality.

##### Global Memory

Participants rated their memory that day (e.g., Today I would rate my own memory as) using a sliding scale from 0 (“Poor”) to 100 (“Excellent”). Higher scores indicate better self-rated global memory each day.

##### Sleep Quality

Participants rated their sleep quality “last night” (e.g., How would you rate the quality of your sleep last night?) on a sliding scale from 0 (“Poor”) to 100 (“Very Good”). Sleep quality represented sleep that occurred the night before and leading into the day participants filled out the form.

### 2.3. Procedure

#### 2.3.1. Pre-Test

Participants completed a remotely administered pre-test session which included consenting, a brief phone interview, and completion of online surveys. During the telephone call, the online forms were reviewed and study staff administered the Telephone Interview for Cognitive Status (TICS) [99]. Generally, a score of 31 or less indicates possible cognitive impairment and is used as an MCI cut-off score [100]. During the telephone call, study staff also conducted a short interview to collect demographic information and a brief medical history. Care recipients and caregivers often scheduled a single phone call but completed their portion of the interview, questionnaires, and TICS independently.

#### 2.3.2. The Nightly Diary

After completing the pre-test session, participants were given an explanation of how to access and complete the nightly diary form entries. Participants were contacted again before the start of their 14-day diary period. The purpose of this check-in was three-fold: (1) as a study reminder, (2) verified participation, and (3) allowed the participants to receive answers to any study-related questions.

Caregivers and care recipients from each dyad completed their own nightly diary form over the same consecutive 14-day time period through Qualtrics. Each evening at approximately five o’clock, each participant received an email with a unique link to that day’s diary form. Participants were given instructions to fill out their responses independently and only on the day specified in the email. If participants missed a day, they were instructed to skip the missed diary and complete the next form with the new link. Participants were contacted after completion of their first diary entry and given the opportunity to ask questions about how to access or fill out any part of the diary. This contact point allowed the study team to verify continued participation in the study. Participant diary data was monitored throughout the study period. If participants missed two or more consecutive diary entries, they were contacted by study personnel. Following the 14-day diary period participants received an email debrief and were thanked for their study participation.

### 2.4. Statistical Analyses

The data was analyzed using multilevel modeling [101]. Analyses were run in R version 4.2.0 [102] using the lme4 package for mixed effects linear modeling [103]. Model predictors at level 1 (day) were all person-mean-centered (i.e., mean-centered across days within individuals). Caregiver burden was investigated as the outcome measure using a series of (concurrent) day models, as well as a series of day-to-next-day (lagged) models using an additive approach. The intraclass correlation coefficient (ICC) was established by running a random intercept-only model. The intercepts were allowed to randomly vary for all models. Models were run with fixed slopes. TICS, age, and education were added as covariates to the intercept-only concurrent and lagged models. Full models included the covariates as well as the predictors of interest and interactions among them. All available diary data were used in the analyses, and missing data were assumed to be missing at random. Diary compliance was high. Caregivers completed 344 total diares with an average of 12.74 (SD = 1.43) diaries per person out of 14 possible. Care recipients completed a total of 325 diaires with an average of 12.04 (SD = 1.89) per person.

The analyses provided information about the magnitude, direction, and statistical significance of each variable’s relationship to the outcome of interest while holding the other variables constant. The full models were then expanded to investigate potential interactions among variables which provided insight on whether and how the relationship among various predictor variables worked together relative to the outcome of interest. All of the models used maximum likelihood estimation for the purpose of model comparison. Final concurrent and lagged models for each hypothesis were selected based on model fit criteria and model specifications through model comparison. Model comparisons along with the selected final best fitting concurrent and lagged models as well as their significant findings are presented below for each hypothesis.

The first goal of data analysis was to identify directional associations among the variables relative to caregiver burden and caregiver depressive affect. The second goal of data analysis was to better understand how different daily life factors were associated with caregiver burden by comparing models to identify the best fitting model. The third goal of data analysis was to evaluate these associations on different time scales based on concurrent (Aim 1) daily occurrences (day-to-day) and day-to-next-day (lagged) occurrences (Aim 2). These analyses allowed us to critically examine whether patternistic associations among these variables that occurred within the same day also showed meaningful influences on next-day variables of interest.

## 3. Results

### 3.1. Concurrent Caregiver Burden

Concurrent models represent individuals’ self-reports over an approximate 24 h period of time for each diary entry beginning with their sleep the night prior and ending when they filled out the diary at the end of each day during the study. For example, Monday’s diary responses contained an individuals’ sleep ratings based on when they went to bed on Sunday night until they woke up on Monday morning.

Daily caregiver stress, caregiver depressive affect, caregiver sleep quality, caregiver mutuality ratings, and care recipient global memory ratings were included as level one model predictors of same-day caregiver burden. Level two predictors included TICS score, age, and level of education. A series of exploratory concurrent models investigating the relationships among these varaibles relative to caregiver burden were run and are described below (see Table 2 for model comparison). Model G was selected as the final model. Model G accounted for more information in the data and contained a three-way interaction that revealed a significant two-way interaction between caregiver depressive affect and care recipient memory ratings compared to similar models. A summary of the fixed and random effects for Model G are presented in Table 3. The conditional pseudo-*R*^2^ (0.53) of Model G indicates that the amount of information accounted for by the fixed and random effects by the model is 52% [104] representing a significant increase from the marginal pseudo-*R*^2^ (0.16).

As expected, caregiver stress was significantly associated with caregiver burden. When all other variables were held constant, for every one unit increase in caregivers’ daily stress ratings, we expect a 0.30 increase in difficulty of caregiver burden ratings (*t* = 5.86, *p* < 0.001) on a 0–100 scale. Mutuality was also significantly associated with same-day caregiver burden. Holding all other variables constant, for every one unit increase in mutuality ratings, we expect a decrease of 13.49 points (out of 100) on ratings of caregiver burden difficulty that same day (*t* = −3.32, *p* < 0.001). Additionally, the two-way interaction of caregiver depressive affect and care recipient memory was also significantly associated with caregiver burden difficulty (*B* = 0.74, *t* = 1.95, *p* = 0.05).

Simple slopes analyses were performed to decompose the interaction. When caregiver depressive affect ratings were more than one standard deviation below an individual’s mean there was a significant negative association between the care recipient’s memory ratings and ratings of caregiver burden difficulty *F*(1282.90) = 3.81, *p* = 0.05. Meaning that on days when caregivers reported much lower depressive affect scores than their average, low care recipient memory ratings were associated with increased difficulty of caregiving.

We hypothesized that higher daily stress and depressive affect in the caregiver, lower mutuality ratings, lower care recipient memory ratings, and lower caregiver sleep quality would be associated with higher caregiver burden within days. Concurrent multilevel analyses revealed significant effects among high daily stress and higher caregiver burden difficulty. Moreover, the analyses revealed that decreases in daily mutuality were significantly associated with same-day decreases in caregiver burden difficulty. Additionally, the significant interaction of caregiver depressive affect and care recipient memory ratings showed that on days when caregivers reported lower than average depressive affect, low care recipient memory ratings were associated with increased caregiver burden difficulty. The concurrent analyses did not reveal significant effects for caregiver sleep on caregiver burden difficulty.

### 3.2. Lagged Caregiver Burden

The lagged models contained the same predictors as the concurrent models with the addition of using the previous day’s caregiver stress and sleep quality ratings (representing sleep from two nights prior) to explore potential day-to-next-day associations. Model F was selected as the final model. It explained the highest amount of information in the data and had a similar model fit to comparable models. Model comparison results are below in Table 4. A summary of the fixed and random effects are presented in Table 5.

The conditional pseudo-*R*^2^ (0.49) of Model F indicates that the model accounts for a significant amount of information in the data compared to the marginal pseudo-*R*^2^ (0.15). Model F had two significant predictors, mutuality and caregiver stress, even while accounting for lagged sleep and stress. Mutuality was negatively associated with caregiver burden difficulty (*t* = 2.63, *p* < 0.001) such that when all other variables are held constant, per one unit increase in mutuality ratings, we expect a 12.55 decrease in caregiver burden difficulty on a 0–100 scale reported that same day. Additionally, caregiver stress was significantly positively associated with same-day caregiver burden difficulty (*t* = 5.62, *p* < 0.001) such that when holding all other variables constant, per one unit increase in caregiver stress ratings, we expect a 0.35 increase in ratings of caregiver burden difficulty on a 0–100 scale reported that same day. None of the other predictor variables were significant, although caregiver depressive affect was trending toward significance (*t* = 1.83, *p* = 0.06).

We expected positive relationships among higher daily stress and depressive affect ratings relative to higher ratings of caregiver burden difficulty. We also expected that there would be lower same-day ratings of caregiver sleep quality, mutuality, and care recipient memory relative to higher caregiver burden difficulty. The lagged analyses revealed a significant positive relationship among caregivers’ daily stress ratings and a significant negative relationship among daily mutuality and caregiver burden when lagged sleep and stress were accounted for by the model. However, these relationships may best be understood within the context of within-day analyses as this pattern of results was also observed in the concurrent model.

### 3.3. Concurrent Caregiver Depressive Affect

Daily ratings of care recipient stress, care recipient depressive affect, caregiver sleep quality, caregiver mutuality, and RMBPC were included as level one model predictors of same-day caregiver depressive affect. Level two predictors remained the same (TICS score, age, and level of education). A series of exploratory models were run that included interactions among the predictor variables and are described below in Table 6. Model E was selected as the final model. Model E showed significant improvement in model fit compared to the full model without interactions and was more parsimonious compared to similar models. The conditional pseudo-*R*^2^ (0.86) was higher than the marginal pseudo-*R*^2^ (0.04) indicating that the model accounts for a large amount of information in the data. Table 7 shows a summary of the fixed and random effects.

Several model predictors were significant. Mutuality was significantly negatively associated with caregiver depressive affect ratings (*t* = −3.86, *p* < 0.001). When all other variables are held constant, for every one-point increase in mutuality ratings we expect that caregiver depressive affect decreases by 0.23 points (out of 100). Moreover, there were four significant two-way interactions. The interaction of care recipient depressive affect and mutuality was significant (*B* = 0.38, *t* = 3.27, *p* < 0.001). The interaction of mutuality and RMBPC scores was significant (*B* = −0.16, *t* = −2.23, *p* = 0.03) as was the interaction of care recipient stress and mutuality (*B* = −0.01, *t* = −2.00, *p* = 0.05). Finally, the interaction of care recipient GDS and RMBPC (*B* = 0.11, *t* = 2.76, *p* = 0.01) was also significant. Simple slopes analyses were performed to decompose these interactions.

#### 3.3.1. Care Recipient Depressive Affect × Mutuality

Daily care recipient depressive affect was significantly associated with mutuality and caregiver depressive affect across days *F*(1276.67) = 10.68, *p* = 0.001. When mutuality scores were one standard deviation or higher than average, there was a significant positive relationship among care recipient depressive affect and caregiver depressive affect. Effectively, on days when mutuality was higher than average, higher depressive affect among care recipients was associated with higher caregiver depressive affect that same day.

#### 3.3.2. Mutuality × RMBPC

Mutuality was associated with RMBPC scores and caregiver depressive affect at the level of day *F*(1277.81) = 4.97, *p* = 0.03. On days when mutuality was more than one standard deviation below an individual’s mean across days, higher RMBPC scores were associated with higher caregiver depressive affect. This relationship flipped when mutuality was more than one standard deviation above an individual’s average, such that there was a negative relationship with RMBPC scores and caregiver depressive affect, meaning they both decreased when mutuality was high.

#### 3.3.3. RMBPC × Care Recipient Depressive Affect

RMBPC was also significantly positively associated with caregiver depressive affect when care recipient depressive affect was approximately one standard deviation above an individual’s mean depressive affect that same day *F*(1276.65) = 7.62, *p* = 0.01. In other words, when caregiver burden was higher and care recipients reported higher depressive affect, caregivers did too.

#### 3.3.4. Care Recipient Stress × Mutuality

Finally, daily care recipient stress ratings were also significantly positively associated with caregiver depressive affect ratings when daily mutuality scores were one standard deviation below individuals’ means *F*(1276.61) = 3.99, *p* = 0.04.

We hypothesized that higher depressive affect in the caregiver would be associated with higher care recipient stress and depressive affect, lower caregiver sleep quality, negative dyadic interaction ratings, and higher caregiver burden via problematic behaviors in care recipients within days. Our hypothesis was largely supported with the exception of caregiver sleep quality. Mutuality emerged as a significant explanatory variable on its own as an independent model predictor and as a moderating factor among daily care recipient RMBPC scores, depressive affect, and stress scores relative to caregiver depressive affect. The results also supported that higher care recipient stress and depressive affect was associated with same-day increased caregiver depressive affect, likely a consequence of emotional contagion. These findings support the important role that relationship dynamics hold with respect to emotional contagion and affective coupling among MCI care recipients and their caregiving spouses at the level of day. Additionally, these results elucidate the complexities associated with MCI caregivers’ daily affective well-being within the context of spouses’ mutuality, depression, caregiver burden (based on problematic behaviors related to cognitive impairment), and stress.

### 3.4. Lagged Caregiver Depressive Affect

The lagged models used the same predictor variables as the full concurrent model described above (without interactions) except lagged caregiver stress and sleep quality ratings were used in place of the concurrent ratings. Caregiver stress was lagged by one day. Sleep was lagged the same way as described in Section 3.2 (by one “diary” day—representing sleep from two nights prior). A series of exploratory models were run; results from model comparison are presented below in Table 8. Model E was selected as the final model. Model E showed the best fit compared to similar models but contained a significant three-way interaction between care recipient stress, mutuality, and lagged caregiver sleep quality. A summary of the fixed and random effects for Model E are in Table 9 below. The conditional pseudo-*R*^2^ (0.90) indicates that the model accounts for a large amount of information in the data.

Same-day care recipient depressive affect and caregiver depressive affect were positively associated. Holding all other variables constant, for every one-point increase in care recipients’ depressive affect ratings, we expect a 0.09 increase (out of four) in caregivers’ depressive affect ratings that same day (*t* = 3.03, *p* < 0.001). Mutuality was significantly negatively associated with caregivers’ depressive affect. Holding all other variables constant, for every one-point increase in mutuality scores, we expect a 0.23 decrease in caregivers’ depressive affect ratings out of four (*t* = −4.89, *p* < 0.001). There were two significant interactions. The first was a two-way interaction between care recipient depressive affect and mutuality (*B* = 0.44, *t* = 3.67, *p* < 0.001). The second was a three-way interaction of care recipient stress, lagged caregiver sleep quality, and mutuality (*B* = 0.00, *t* = 2.31, *p* = 0.02). The two-way interaction of lagged caregiver stress and mutuality was trending toward but did not reach significance. Simple slopes analyses were run to decompose the significant two- and three-way interactions.

#### 3.4.1. Care Recipient Depressive Affect × Mutuality

For the two-way interaction of care recipient depressive affect and mutuality, on days when mutuality was approximately an individual’s mean or higher, there was a significant positive association between care recipient depressive affect and caregiver depressive affect *F*(1232.18) = 13.46, *p* < 0.001. Meaning that on days when mutuality was approximately average or higher, higher caregiver and care recipient depressive affect co-occurred that same day.

#### 3.4.2. Care Recipient Stress × Lagged Caregiver Sleep Quality × Mutuality

For the significant three-way interaction, when caregivers’ lagged sleep quality (two nights prior) was approximately one standard deviation below an individual’s mean and next-day (two days later or one “diary” day later) mutuality was approximately one standard deviation below an individual’s mean, there was a positive significant association between that day’s care recipient stress and caregiver depressive affect (yellow line far left graph) *F*(1231.47) = 5.33, *p* = 0.02. Moreover, when lagged caregiver sleep quality was one standard deviation below an individual’s average and mutuality was approximately one standard deviation above an individual’s mean, there was a significant negative association between same-day care recipient stress and caregiver depressive affect (yellow line, far right graph). Meaning, when caregivers had a sleep debt from lower sleep quality two nights prior, the relationship between same-day care recipient stress and caregiver depressive affect co-occurred two days later, in that they both increased when mutuality was lower than normal–a potential contagion effect. However, when caregivers had a sleep debt from two nights prior and two days later mutuality was higher than average, caregiver depressive affect was not reflective of higher care recipient stress that same day. Essentially, higher relationship mutuality moderated the effect of one spouse’s stress increasing the other’s depressive affect when lagged sleep quality was accounted for in the model.

We hypothesized that higher depressive affect in the caregiver would be associated with higher care recipient stress and depressive affect, lower caregiver sleep quality, negative dyadic interaction ratings, and higher caregiver burden from one day to the next. When lagged caregiver sleep quality and stress were accounted for in the multilevel models, our hypothesis was largely supported. Care recipient depressive affect was significant over and above the interaction effects. This finding is another example of the importance of depression displaying a contagion effect among MCI care recipient and caregiver spousal dyads. Moreover, the pervasive effects of mutuality were significant, not only in the significant two- and three-way interactions but also as an independent predictor of caregiver depressive affect over and above its role in the interactions. Mutuality seems to act as a buffer against depressive affect and stress contagion between spouses. Additionally, the effect of sleep debt was identified as a potential vulnerability for spousal MCI caregivers. Poorer sleep quality may compound by building up sleep debt within an individual over time. A sleep debt in caregivers from two nights prior was associated with increased depressive affect and stress contagion effects from their care recipient two days later. Finally, the three-way interaction of poor lagged caregiver sleep quality relative to mutuality and stress revealed that on days when caregivers had a sleep debt from two nights prior and mutuality was below average, as care recipient stress increased so did caregiver depressive affect. However, that relationship flipped and the contagion effect was not present for caregivers’ depressive affect when mutuality was above average even when caregivers had a sleep debt from two nights prior and care recipient stress was high—showcasing mutuality’s moderating role in protecting caregivers’ affective well-being (i.e., depressive affect).

## 4. Discussion

MCI caregiving is significant, complex, and nuanced compared to other caregiving contexts. The present work sought to investigate MCI caregiving among spousal dyads using a highly contextualized approach, encompassing aspects of daily living and caregiving from spousal caregivers and care recipients with MCI. We expand on current daily diary work in several important ways. Our innovative methodology reveals new insights about contagion effects and the relevance of including data from both dyadic partners. Further, our concurrent and lagged analyses underscore the need to account for temporal dynamics associated with contagion across sleep debt, mutuality, depressive affect, and stress. Our findings highlight the importance of contextual factors that impact the well-being and daily lived experiences of spousal MCI caregivers, including their care recipient, spousal contagion, and relationship mutuality. Our study findings present novel outcomes that meaningfully contribute to the literature on MCI caring dyads’ everyday lived experiences.

Adapting the stress process model framework to the present investigation, indicators of primary stressors included TICS score, memory ratings, behavioral problems as reported by the RMBPC, and ratings of caregiving difficulty. Secondary stressors included ratings of non-caregiving related variables, namely, education, age, depressive affect, stress, and sleep quality ratings. Mutuality played a moderating role as a buffer between the primary and secondary stressors. The pervasive significance of mutuality as an explanatory variable across the concurrent and lagged hypotheses presents a potential opportunity to expand the caregiving stress process model in MCI by including mutuality as an important moderator. The bolstering and deleterious effects of mutuality on caregiver burden and depressive affect act in a similar fashion to coping (e.g., laughing together) and social support (love for one another) which both serve as mediators in the original stress process model framework. Although we did not evaluate mutuality as a mediator in this instance, its role as a moderator is intriguing since the Mutuality Scale includes aspects of social support and coping. Mutuality appears to be a promising way to contextualize both the positive and negative aspects of relationship dynamics among spousal dyads impacted by MCI.

As stated, mutuality was significantly associated with caregiver burden and depressive affect outcomes among caregivers across the concurrent and lagged hypotheses, appearing to buffer potential negative impacts of care recipients’ problematic behaviors, depressive affect, and global stress. For example, when mutuality was higher than average, caregiver depressive affect was not reflective of higher care recipient stress when caregivers had a sleep debt. Additionally, when lagged sleep was accounted for, high relationship mutuality buffered the contagion effect of care recipients’ stress increasing caregivers’ depressive affect. However, on days when care recipients had more problematic behaviors and reported higher depressive affect, caregivers also reported higher depressive affect. Moreover, care recipient depressive affect and caregiver depressive affect were positively associated when mutuality was higher than daily averages. Each displayed a different pattern of results but mutuality was significant across each of our study aims and hypotheses, again pointing to its importance in influencing daily and day-to-next-day caregiver outcomes.

Within an MCI caregiving context, the role of mutuality may be connected to changing relationship dynamics at a highly uncertain point in time for caregivers and care recipients alike. MCI is an extremely heterogenous condition and is not merely a transitional state between normal cognition and dementia [105]. The uncertainty of MCI progression into dementia also impacts spouses who may or may not transition from informal MCI caregivers to dementia caregivers if their spouse’s cognition declines in the future and requires a higher level of care. An MCI diagnosis represents an intervention opportunity not only for the benefit of spousal MCI caregivers but for the benefit of the care recipient as well. Enhancing aspects of relationship dynamics through mutuality presents very real intervention targets with the potential to positively enhance daily living for spouses impacted by MCI. Whether the care recipient with MCI will revert back to normal cognitive status, remain stable in MCI, or progress to a dementia diagnosis, enhancing the relationship dynamics of impacted spouses and decreasing contagion effects has the potential to create lasting positive impacts on marital satisfaction and perceived quality of life for both spouses [42,56].

Additionally, although the use of the RMBPC to capture cognition-related behavioral problems in the care recipient and the ratings of caregiving difficulty are not wholly representative of experienced caregiver burden, independently they showed a variety of interesting results. RMBPC has been used in the literature as a proxy measure of caregiver burden. It is an indirect representation of potential burden and is a rating about the care recipient rather than the caregiver, hence its use as a predictor variable in the present study. In the models investigating caregiver depressive affect, caregiver burden was measured via care recipients’ behaviors relative to both dyad members’ internal states (i.e., depressive affect, stress). Investigating caregiver burden through RMBPC does not directly capture caregivers’ internal states that reflect how these individuals feel about caregiving and does not holistically capture MCI caregivers’ daily experiences. However, it does provide useful context about the impact of care recipients’ behaviors on mutuality and contagion effects among spousal dyads. In contrast, the difficulty of caregiving rating was a novel outcome measure representing caregiver burden based on caregiving circumstances and subjective difficulty. Direct comparisons of internal states (e.g., depressive affect, stress) within individuals and between spouses were examined. Caregiving difficulty ratings provided unique insight on caregiver burden through daily internal experiences and were then contextualized based on associations with other secondary stressors from caregivers and their care recipients. The pattern of results supports and extends prior work on contagion among spouses for daily depression and stress ratings [40,42,47,52,53] by including a wide array of everyday influences and by investigating concurrent and lagged analyses. Future investigations should investigate daily caregiver burden via multiple measures to capture various aspects of the caregiving experience (e.g., RMBPC, how rewarding caregiving was, physical and mental health of the caregiver).

### 4.1. Limitations

The present study has several important limitations to consider. The sample used in this study is not necessarily representative of the population of older adults with diagnosed MCI nor their spousal caregivers. The sample was drawn exclusively from a single site, the CEP, and not the greater Atlanta community. The majority of participants in this study were well-educated and white. Importantly, the CEP is a therapeutic research program for those with diagnosed MCI and their caregivers which provides brain healthy lifestyle education and access to resources across the domains of physical activity, cognitive strategies, home and personal safety, emotional well-being, and nutrition. The breadth and depth of education and resources available to dyads in the CEP is not the norm for older adults diagnosed with MCI and their caregivers. Thus, it is possible that participation in CEP impacts the factors assessed in this study which limits the generalizability of our findings. Additionally, the lack of adequate demographic representation limits the generalizability of our findings. Nevertheless, our results highlight the value of our innovative approach and provide insight to guide future multidimensional caring dyad research. Future investigations should consider a larger, more representative, diverse sample of diagnosed MCI persons and their spousal caregivers.

The present work also used self-reported data from both MCI caregivers and care recipients with MCI. Capturing a holistic understanding of MCI caregiver burden and depressive affect within the context of daily stress, spousal relationship dynamics, memory functioning, and sleep cannot be objectively ascertained from self-report measures alone, which may be subject to recall bias. Subjective ratings of individuals daily lived experiences fits the study aims—understanding within day and day-to-next-day patterns across affective well-being, caregiving, stress, relationship dynamics, cognition, and sleep. However, individuals, and especially those with MCI, may not always be the best judge of things like their sleep quality compared to objective measures (i.e., actigraphy). Multidomain approaches should be used to truly understand and measure caring dyads’ contextually relevant daily experiences, behaviors, internal states, sleep, and dyadic factors. Importantly, however, individuals’ perspectives about their daily lives matter and patients’ perspectives play a critical role in motivation and behavior change [106,107]. Nonetheless, subjective self-reports should also ideally be accompanied by objective measures to ensure that the information gained is unbiased and meaningful in shaping future interventions for caring dyads. Finally, we are limited by the use of repeated measures from partial, unvalidated scales among our study sample of caring MCI dyads. Partial scales (e.g., Mutuality, RMBPC) were intentionally shortened and we used single 0–100 ratings for key variables (e.g., sleep quality, cognition, stress) to reduce participant burden. Our findings should be interpreted with these limitations in mind.

### 4.2. Future Directions

Future investigations that build on the present study should consider alternative time course data collection approaches. For example, future studies could consider an ecological momentary assessment approach to gather data at multiple points throughout each day to understand within-day variability with more acuity. Additionally, alternative approaches, such as a burst longitudinal study design could capture daily diary data several times throughout the course of a year—or longer. These approaches could also incorporate objective measures using actigraphy to measure sleep and other key constructs alongside diary self-reports. Measurement periods could then be aligned with various diagnostic or life events to better hone-in on stability or change within and across individuals and dyads over a longer period of time and across various measures of cognition, well-being, and functioning.

Additionally, future studies should also investigate key components of mutuality in caring MCI dyads that may inform interventions. Resources, strategies, and approaches that can enhance dyadic mutuality and reduce negative spousal contagion in MCI may impact daily well-being and functioning but remain to be identified and tested. Interventions seeking to enhance mutuality may be helpful for both members of the dyad, but especially for caregivers’ experienced daily burden and depressive affect. Popular caregiver interventions often target problem solving, e.g., [108], or decision-making [109,110]. From the lens of mutuality, perhaps other avenues through practices that serve to enhance shared pleasurable experiences, reciprocity through positive communication strategies for expressions of love or one’s affective needs, or gentle reminders regarding cognitive problems or safety may be potential intervention options [111,112].

Other factors that could be taken into consideration for interventions include management of stress and sleep disruption. Stress management techniques and activities that decrease depressive affect for both spouses may also enhance the daily lived experiences and well-being of these dyads by buffering contagion [38,50]. Finally, the role of sleep debt and drivers of poor sleep quality (i.e., sleep disturbances) among caregivers should also be a focus of future research as it may fuel contagion. Caregivers were more vulnerable to negative contagion effects when they experienced sleep debt from poor quality sleep two nights prior (even when their most recent night’s sleep quality was rated as high). Improving sleep hygiene may be a potent intervention with additional reciprocal effects across a myriad of factors for MCI caring dyads.

## 5. Conclusions

This novel study investigated caregiver burden from a holistic, contextually relevant lens for caring dyads. We elucidated key findings regarding the complex nature of caregiver burden and depression outcomes for spousal MCI caregivers based on daily fluctuations in caregiver burden, depressive affect, stress, mutuality, cognition, and sleep within days and from one day to the next. Mutuality emerged as a double-edged sword and topic of future research interest, serving as multifaceted moderator in both protecting against and enhancing the deleterious effects of stress and depressive affect contagion among spousal dyads. Ultimately, the relationship between spousal MCI caregivers and their care recipients plays a pivotal role in determining caregivers’ day-to-day outcomes.

## Figures and Tables

**Table 1:** Caregiver and care recipient demographics.

	Caregivers	Care Recipients
Mean Age	71.4 (8.35)	74.6 (8.26)
Gender	18 Female	10 Female
Mean Education	17.10 (2.14)	16.62 (2.62)
Mean TICS Score	36.19 (2.96)	29.81 (4.96)
Mean Medications	3.56 (2.14)	4.92 (2.22)
Mean Conditions	4 (3)	5 (3)
Percent Caucasian	96.3%	85.19%

Note: Numbers in parentheses indicate the standard deviation of the mean. Mean medications refers to the average total number of prescription medications participants indicated they were taking at the time of study enrollment. Mean conditions refers to the average number of current health conditions participants reported. Percent Caucasian refers to the percent of participants in who identified as Caucasian.

**Table 2:** Concurrent caregiver burden model comparison.

Model (Predictors)	ICC	*n* Parameters	AIC	BIC	−2LL	Chi-Square (df)	*p*
A: (Intercept-only)	0.35	3	3046.7	3058.2	3040.7	--	--
B: (Model A + TICS + Age + Edu)	0.32	6	3049.4	3072.5	3037.4	A: 3.22 (3)	0.36
C: (Model B + CG Stress + CG GDS + CG Sleep + CR Memory + Mutuality)	0.42	11	2566.4	2606.9	2544.4	B: 493 (5)	<0.05 *
D: (Model C + CG GDS * CR Memory	0.44	12	2565.3	2609.5	2541.3	C: 3.10 (1)	0.08
E: (Model C + CG Sleep * CG Stress)	0.42	12	2567.2	2611.4	2543.2	D: 0.10 (0)	1.00
F: (Model D + CG Stress * CR Memory)	0.43	13	2565.6	2613.5	2539.6	E:3.34 (1)	0.05 *
G: (Model F + Mutuality * CRMemory * CG GDS	0.44	16	2568.3	2627.3	2536.3	F: 3.26 (3)	0.35

Note: CG denotes caregiver data; CR denotes care recipient data; −2LL = −2 * log likelihood; * = significant for *p*-values ≤ 0.05. Model G was selected as the final model.

**Table 3:** Concurrent caregiver burden Model G summary.

Fixed Effects	Estimate (*B*)	S.E.	T-Val	DF	*p*
Intercept	−9.75	60.03	−0.16	26.52	0.87
TICS	−0.41	1.06	−0.38	26.16	0.71
Age	0.42	0.38	1.10	26.50	0.28
Education	1.17	1.39	0.84	25.48	0.41
CG Stress	0.30	0.05	5.86	267.45	0.00 *
CG GDS	3.78	4.29	0.88	269.41	0.38
Mutuality	−13.49	4.06	−3.32	269.32	0.00 *
CR Memory	−0.05	0.08	−0.66	268.28	0.51
CG Sleep Quality	0.03	0.06	0.50	267.16	0.62
Mutuality * CR Memory	0.25	0.35	0.74	269.94	0.46
CG GDS * Mutuality	5.86	11.33	0.52	284.77	0.61
CG GDS * CR Memory	0.74	0.38	1.95	282.90	0.05 *
CG Stress * CR Memory	0.01	0.00	1.24	280.19	0.21
CG GDS * Mutuality * CR Memory	0.47	0.45	1.04	273.21	0.30
Random Effects					
Parameter	Variance	SD			
Intercept	200.80	14.17			
Residual	259.10	16.10			
Model Information	Pseudo-*R*^2^ Fixed	Pseudo-*R*^2^ Total			
ICC = 0.44	0.16	0.53			

Note: * = significant for *p*-values ≤ 0.05, Pseudo-*R*^2^ Fixed represents the marginal *R*^2^ (amount of information accounted for by the fixed effects in the model), and Pseudo-*R*^2^ Total represents the conditional *R*^2^ (the amount of information accounted for by the fixed and random effects in the model). Significant model predictors were: caregiver stress, mutuality, and the two-way interaction of caregiver GDS × care recipient memory. Higher mutuality was associated with decreased caregiver burden and lower than average caregiver depressive affect was associated with increased difficulty of caregiving when care recipient memory ratings were low.

**Table 4:** Lagged caregiver burden model comparison.

Model (Predictors)	ICC	*n* Parameters	AIC	BIC	−2LL	Chi-Square (df)	*p*
A: (Intercept-only)	0.35	3	3046.7	3058.2	3040.7	--	--
B: (Model A + TICS + Age + Edu)	0.32	6	3049.4	3072.5	3037.4	A: 3.22 (3)	0.36
C: (Model B + CGL Stress + CG GDS + CGLSQ+ CR Memory + Mutuality)	0.36	11	2226.8	2265.7	2204.8	B: 832.6 (5)	<0.05 *
D: (Model C + CG Stress)	0.40	12	2200.7	2243.0	2176.7	C: 28.17 (1)	<0.05 *
E: (Model D + CGLSQ * CG Stress)	0.40	13	2201.4	2247.2	2175.4	D: 1.30 (1)	0.25
F: (Model D + CG Stress * CGLSQ)	0.40	13	2201.4	2247.2	2175.4	E:0.00 (0)	1.00

Note: CGL Stress = lagged CG stress, CGLSQ = lagged CG sleep quality, −2LL = −2 * log likelihood, * = significant for *p*-values ≤ 0.05. Model F was selected as the final model.

**Table 5:** Lagged caregiver burden Model F summary.

Fixed Effects	Estimate (*B*)	S.E.	T-Val	DF	*p*
Intercept	−29.50	58.76	−0.50	28.25	0.62
Age	0.57	0.37	1.54	28.05	0.14
Education	1.16	1.34	0.86	26.13	0.40
TICS	−0.16	1.03	−0.16	27.53	0.87
CGL Stress	−0.08	0.05	−1.51	227.83	0.13
CG GDS	9.09	4.91	1.83	228.67	0.06
CGLSQ	0.05	0.07	0.63	229.57	0.53
CR Global Memory	−0.04	0.08	−0.46	227.07	0.65
Mutuality	−12.55	4.77	−2.63	230.31	0.00 *
CG Stress	0.35	0.06	5.62	227.88	0.00 *
CGLSQ * CG Stress	0.00	0.00	1.14	228.51	0.25
Random Effects					
Parameter	Variance	SD			
Intercept	181.4	13.47			
Residual	266.9	16.34			
Model Information	Pseudo-*R*^2^ Fixed	Pseudo-*R*^2^ Total			
ICC = 0.40	0.15	0.49			

Note: * = significant for *p*-values ≤ 0.05, Pseudo-*R*^2^ Fixed represents the marginal *R*^2^ (amount of information accounted for by the fixed effects in the model), and Pseudo-*R*^2^ Total represents the conditional *R^2^* (the amount of information accounted for by the fixed and random effects in the model). Two significant predictors were identified, mutuality and caregiver stress, when accounting for lagged caregiver sleep quality and stress. Increased mutuality was associated with decreased caregiver burden difficulty. Increased ratings of caregiver stress were associated with increased ratings of caregiver burden difficulty.

**Table 6:** Concurrent caregiver depressive affect model comparison.

Model (Predictors)	ICC	*n* Parameters	AIC	BIC	−2LL	Chi-Square (df)	*p*
A: (Intercept-only)	0.76	3	266.84	278.36	260.84	--	--
B: (Model A + TICS + Age + Edu)	0.76	6	272.46	295.50	260.45	A: 0.38 (3)	0.94
C: (Model B + CR Stress + CR GDS + CG Sleep + Mutuality + RMBPC)	0.84	11	165.91	206.76	143.91	B: 116.54 (5)	<0.05 *
D: (Model C + Mutuality * CR GDS + Mutuality * RMBPC + Mutuality * CG Sleep)	0.85	14	158.43	210.43	130.43	C: 13.47 (3)	<0.05 *
E: (Model C + Mutuality * RMBPC * CR GDS + CR Stress * Mutuality)	0.85	16	150.03	209.44	118.03	D: 12.41 (2)	<0.05 *
F: (Model C + Mutuality * CR GDS + Mutuality * RMBPC * CG Sleep + CR Stress * Mutuality)	0.85	17	159.56	222.69	125.56	E: 0.00 (1)	1.00

Note: −2LL = −2 * log likelihood; * = significant for *p*-values ≤ 0.05. Model E was selected as the final model.

**Table 7:** Concurrent Caregiver Depressive Affect Model E Summary.

Fixed Effects	Estimate (*B*)	S.E.	T-Val	DF	*p*
Intercept	1.38	2.35	0.59	26. 89	0.56
TICS	−0.02	0.04	−0.39	26.86	0.70
Age	−0.01	0.02	−0.39	26.96	0.70
Education	−0.01	0.05	−0.21	26.82	0.83
CG Stress	0.00	0.00	0.85	275.90	0.40
CR GDS	0.04	0.03	1.37	275.89	0.17
CG Sleep Quality	0.00	0.00	0.02	276.89	0.98
Mutuality	−0.23	0.06	−3.86	276.08	0.00 *
RMBPC	0.01	0.02	0.45	276.00	0.66
Mutuality * RMBPC	−0.16	0.07	−2.23	277.81	0.03 *
CR GDS * Mutuality	0.38	0.12	3.27	276.67	0.00 *
CR GDS * RMBPC	0.11	0.04	2.76	276.65	0.01 *
CR Stress * Mutuality	−0.01	0.00	−2.00	276.61	0.05 *
CR GDS * Mutuality * RMBPC	0.10	0.11	0.92	276.49	0.36
Random Effects					
Parameter	Variance	SD			
Intercept	0.35	0.59			
Residual	0.06	0.24			
Model Information	Pseudo-*R*^2^ Fixed	Pseudo-*R*^2^ Total			
ICC = 0.85	0.04	0.86			

Note: * = significant for *p*-values ≤ 0.05. Pseudo-*R*^2^ Fixed represents the marginal *R*^2^ (amount of information accounted for by the fixed effects in the model) and Pseudo-*R*^2^ Total represents the conditional *R*^2^ (the amount of information accounted for by the fixed and random effects in the model). Mutuality was a significant model predictor, along with the following two-way interactions: mutuality × RMBPC, care recipient GDS × mutuality, care recipient GDS × RMBPC, and care recipient stress × mutuality. Increased mutuality was associated with decreased caregiver depressive affect. Higher care recipient stress and depressive affect was associated with increased caregiver depressive affect.

**Table 8:** Lagged caregiver depressive affect model comparison.

Model (Predictors)	ICC	*n* Parameters	AIC	BIC	−2LL	Chi-Square (df)	*p*
A: (Intercept-only)	0.76	3	266.84	278.36	260.84	--	--
B: (Model A + TICS + Age + Edu)	0.76	6	272.46	295.50	260.45	A: 0.38 (3)	0.94
C: Model B + CR Stress + CGL Stress + CR GDS + CGLSQ + Mutuality + RMBPC)	0.88	12	101.13	143.77	77.13	B: 183.32 (6)	<0.05 *
D: (Model C + Mutuality * CR GDS + Mutuality * RMBPC + CR Stress * RMBPC)	0.89	17	91.88	152.28	57.88	C: 19.25 (5)	<0.05 *
E: (Model C + Mutuality * CR GDS + CGL Stress * Mutuality + CR Stress * CGLSQ * Mutuality)	0.89	18	90.37	154.32	54.37	D: 3.51 (1)	0.06
F: (Model C + Mutuality * CR GDS + Mutuality * RMBPC + CR Stress * RMBPC + CGLSQ * Mutuality + CGLSQ * CR stress + CGLSQ * CR GDS)	0.89	18	97.51	97.51	61.51	E: 0.00 (0)	1.00

Note: CGL Stress = Lagged CG Stress, CGLSQ = lagged CG Sleep Quality, −2LL = −2 * log likelihood, * = significant for *p*-values ≤ 0.05. Model E was selected as the final model.

**Table 9:** Lagged caregiver depressive affect Model E summary.

Fixed Effects	Estimate (*B*)	S.E.	T-Val	DF	*p*
Intercept	0.94	2.45	0.38	26.66	0.70
TICS	−0.01	0.04	−0.16	26.62	0.87
Age	−0.00	0.02	−0.27	26.67	0.79
Education	−0.01	0.06	−0.22	26.55	0.83
CR Stress	0.00	0.00	0.21	230.70	0.83
CGL Stress	−0.00	0.00	−0.29	230.72	0.78
CR GDS	0.09	0.03	3.03	230.83	0.00 *
CGLSQ	0.00	0.00	1.09	231.07	0.28
Mutuality	−0.32	0.06	−4.89	231.41	0.00 *
RMBPC	−0.01	0.02	−0.31	231.00	0.76
CR GDS * Mutuality	0.44	0.12	3.67	232.18	0.00 *
CGL Stress * Mutuality	−0.01	0.00	−1.88	231.06	0.06
CR Stress * CGLSQ	0.00	0.00	0.74	231.48	0.46
CR Stress * Mutuality	−0.01	0.00	−1.76	231.21	0.08
CGLSQ * Mutuality	0.01	0.01	0.89	231.52	0.37
CR Stress * CGLSQ * Mutuality	0.00	0.00	2.31	231.47	0.02 *
Random Effects					
Parameter	Variance	SD			
Intercept	0.38	0.62			
Residual	0.04	0.21			
Model Information	Pseudo-*R*^2^ Fixed	Pseudo-*R*^2^ Total			
ICC = 0.85	0.03	0.90			

Note: * = significant for *p*-values ≤ 0.05. Pseudo-*R*^2^ Fixed represents the marginal *R*^2^ (amount of information accounted for by the fixed effects in the model) and Pseudo-*R*^2^ Total represents the conditional *R*^2^ (the amount of information accounted for by the fixed and random effects in the model). Significant model predictors were as follows: care recipient GDS, mutuality, the two-way interaction of care recipient GDS × mutuality, and the three-way interaction of care recipient stress × caregiver lagged sleep quality × mutuality. When lagged caregiver sleep quality and stress were accounted for, increased mutuality was associated with decreased caregiver depressive affect. Contagion effects emerged for caregiver and care recipient depressive affect ratings when mutuality was not lower than average. Higher mutuality moderated care recipient stress contagion for caregiver depressive affect when caregivers experienced sleep debt.

## Data Availability

The data presented in this study are available upon request to the corresponding author.
